# 3D Printed Platform for Impedimetric Sensing of Liquids and Microfluidic Channels

**DOI:** 10.1021/acs.analchem.2c03191

**Published:** 2022-10-06

**Authors:** Táňa Sebechlebská, Eva Vaněčková, Marta Katarzyna Choińska-Młynarczyk, Tomáš Navrátil, Lukasz Poltorak, Andrea Bonini, Federico Vivaldi, Viliam Kolivoška

**Affiliations:** † Department of Physical and Theoretical Chemistry, Faculty of Natural Sciences, Comenius University in Bratislava, Mlynska Dolina, Ilkovicova 6, 84215Bratislava 4, Slovakia; ‡ J. Heyrovsky Institute of Physical Chemistry of the Czech Academy of Sciences, Dolejskova 3, 18223Prague, Czech Republic; § Department of Inorganic and Analytical Chemistry, Faculty of Chemistry, University of Lodz, Tamka 12, 91-403Lodz, Poland; ∥ Department of Chemistry and Industrial Chemistry, University of Pisa, via Giuseppe Moruzzi 13, 56124Pisa, Italy

## Abstract

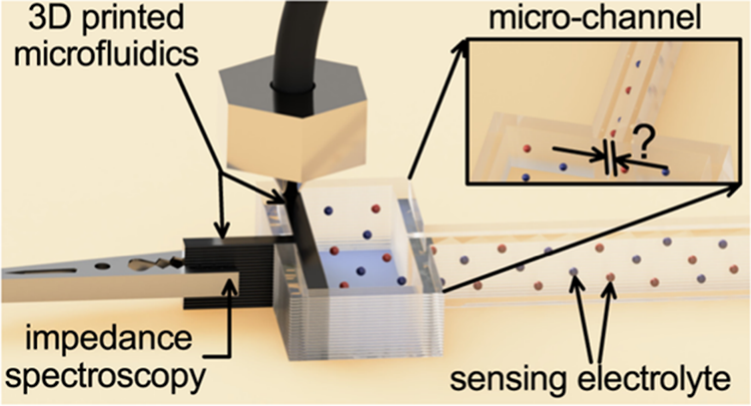

Fused deposition modeling 3D printing (FDM-3DP) employing electrically conductive filaments has recently been recognized as an exceptionally attractive tool for the manufacture of sensing devices. However, capabilities of 3DP electrodes to measure electric properties of materials have not yet been explored. To bridge this gap, we employ bimaterial FDM-3DP combining electrically conductive and insulating filaments to build an integrated platform for sensing conductivity and permittivity of liquids by impedance measurements. The functionality of the device is demonstrated by measuring conductivity of aqueous potassium chloride solution and bottled water samples and permittivity of water, ethanol, and their mixtures. We further implement an original idea of applying impedance measurements to investigate dimensions of 3DP channels as base structures of microfluidic devices, complemented by their optical microscopic analysis. We demonstrate that FDM-3DP allows the manufacture of microchannels of width down to 80 μm.

## Introduction

Recent years have seen a boom in the utilization of 3D printing (3DP) technologies in a broad range of sensing applications. The use of fused deposition modeling (FDM) as the most common 3DP technique has been expanded thanks to the development of electrically conductive filaments. Such materials are based on thermoplastic binders (typically polylactic acid, PLA, or acrylonitrile butadiene styrene, ABS) blended with electrically conductive additives such as graphene (G), carbon black, or carbon nanotubes (CNTs). Objects printed from these composites found applications as sensors to monitor environment stimuli including temperature,^1^ various chemicals,^2,3^ or stress and strain.^4,5^ Investigated stimuli were quantified based on changes in the bulk electric conductance of the sensing object. Carbon-based 3DP electrodes were further shown to have almost ideal charge transfer characteristics^6−11^ and were employed in electrochemical sensing devices to determine diverse analytes involving heavy metals,^6,12−16^ carbon dioxide,^17^ nitrite,^18^ hydrogen peroxide,^19^ glucose,^18,20^ dopamine,^7,21^ uric acid,^18,21^ ascorbic acid,^21,22^ dipyrone,^7^ diclofenac,^7^ catechol,^7,8^*tert*-butylhydroquinone,^7^ 2,4,6-trinitrotoluene,^23^ and picric acid.^22^ 3DP electrodes further found applications in pH sensing,^24^ spectroelectrochemical analysis,^9^ electrosynthesis,^25^ electrochemical water splitting,^26^ and carbon dioxide reduction.^27^

Electric conductivity κ and dielectric permittivity ϵ_r_ are fundamental material characteristics. Their sensing finds use in many applications including the assessment of drinking water quality, conductometric titrations, or characterization of newly synthesized compounds. Several studies have reported the determination of electric conductivity in 3DP cells, all of them utilizing conventional metallic electrodes as sensing elements.^28−31^ Duarte et al. utilized bimaterial printing employing insulating ABS and conductive PLA/CNT to manufacture a microfluidic junction equipped with sensing electrodes functioning as a capacitively coupled contactless conductivity detector. The apparatus was employed to determine the size of oil and water microdroplets^32^ and count *Escherichia coli* cells.^33^ Radonic et al.^34^ reported measurements of relative permittivity of toluene/methanol mixtures in 3DP microfluidic channels equipped with aluminum sensing electrodes.

**Figure 1:**
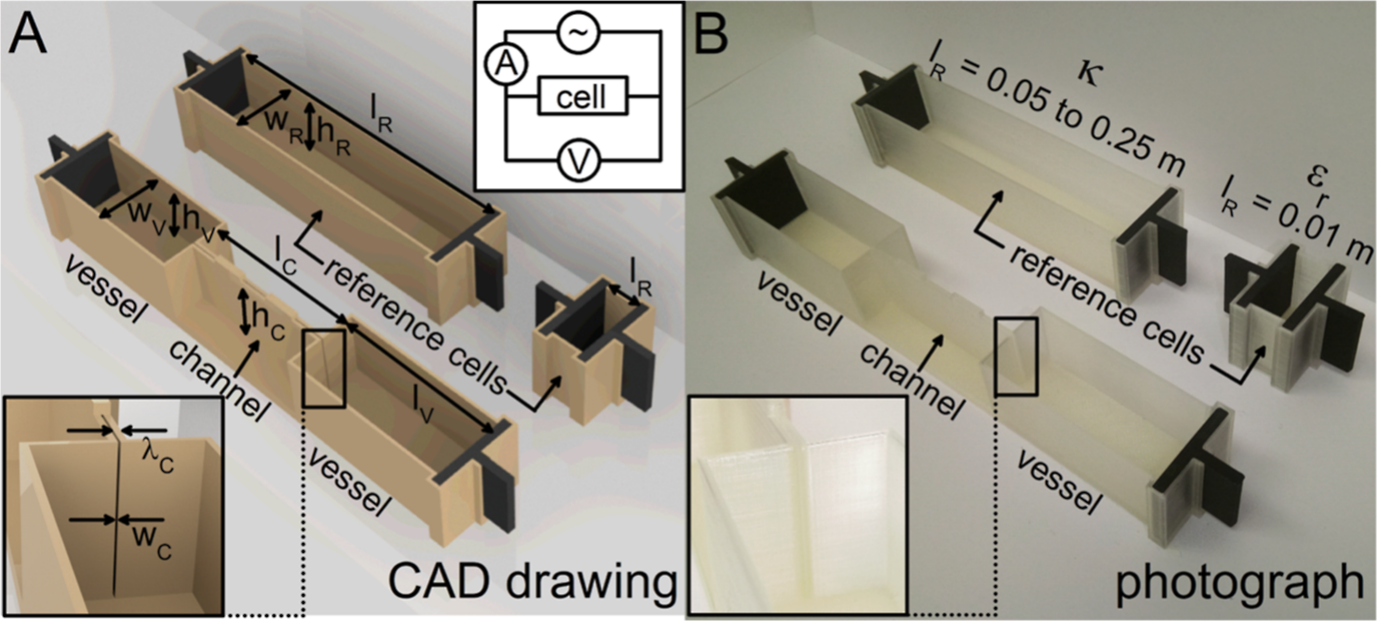
(A) CAD drawing and (B) photograph of a cell involving a microchannel (*l*_C_ = 0.05 m and *w*_C_ = 300 μm, left) and reference cells for κ and ϵ_r_ measurements (right). Insets show the detail of the microchannel and the scheme of the measurement circuit utilized in this work.

The applicability of 3DP electrodes in direct sensing of electric properties of liquids has not yet been explored. To bridge this gap, we utilize bimaterial FDM-3DP combining insulating PLA and conductive PLA/CNT filaments to build an integrated platform for sensing κ and ϵ_r_ based on impedance measurements (the configuration referred to as reference cells, see Figure 1). The platform is based on a rectangular cell with sensing electrodes (denoted as black) in a parallel-plate configuration, enabling uncomplicated conversion of impedance data to desired characteristics. The functionality of the device is demonstrated by determining κ of aqueous potassium chloride solution and bottled water samples and ϵ_r_ of water/ethanol mixtures.

Efforts to reduce the consumption of samples and reagents in sensing have resulted in the development of microfluidic lab-on-a-chip devices. Technologies based on 3DP have proven to be particularly attractive for creating microscopic features.^35−37^ Structures common to all microfluidic devices are channels as conduits for the transport of liquids. Stereolithography (SLA) and digital light processing (DLP) 3DP are based on hardening resins using a precisely targeted UV beam. Recent developments in these techniques allowed substantial miniaturization of channels with cross-sectional dimensions reaching values below 200 μm.^38−44^ However, objects printed by SLA and DLP must be extensively rinsed by organic solvents to remove unreacted resins from their interior. Such a post-processing step is not needed for FDM-3DP, which creates channels as voids in the pattern of the extruded material. The vast majority of studies utilizing FDM-3DP to create channels report their dimensions in the range of hundreds of micrometers.^37^ Only three studies succeeded at making channels with cross-sections below 200 μm.^45−47^ This indicates that capabilities of FDM-3DP in the field of microfluidics have been underexplored.

Real dimensions of 3DP channels were in the above-cited studies determined by optical microscopy imaging. For non-translucent materials, the optical inspection of the channel interior requires its disintegration. Furthermore, such an approach does not provide information on the channel functionality (mass transport, electric conductance, etc.). Here, we introduce a non-destructive experimental approach to determine dimensions of microchannels integrated into the devised measurement platform (Figure 1) based on impedance measurements employing an aqueous sensing electrolyte. Kozlov and Fadina developed a comprehensive theoretical model to predict impedimetric response of microchannels filled with electrolytes.^48^ However, to the best of our knowledge, there is no experimental work utilizing impedance measurements to sense dimensions of microarchitectures. We complement the impedimetric sensing of microchannels by optical microscopic imaging of silicone imprints hardened in their interior. Following the approach of Mehta et al.,^49^ we tune the value of the extrusion multiplier in the 3DP protocol and demonstrate that microchannels of width down to 80 μm may be manufactured. We further build a theoretical model relating the microchannel width and the extrusion multiplier and compare the predicted dependence to experimental results.

## Experimental Section

### Design of Cells and Electrodes

Measurements of κ and ϵ_r_ of liquid samples were performed in platforms denoted as reference cells (see Figure 1A,B, right). Additionally, microchannels were integrated into cells (see Figure 1A,B, left), and their dimensions were further inspected as follows. Both types of cells, together with sensing electrodes (shown as black), were devised in Autodesk Fusion 360 (Autodesk Inc., USA) computer assisted design (CAD) software. Reference cells have a constant inner height *h*_R_ and width w_R_ (both 0.02 m). For conductivity measurements, their length *l*_R_ ranges from 0.05 to 0.25 m in increments of 0.05 m. For permittivity measurements, the *l*_R_ value is constant (0.01 m). Cells involving microchannels contain two identical side vessels with a constant height *h*_v_ of 0.02 m, a width *w*_v_ of 0.02 m, and a length *l*_v_ of 0.05 m. Dimensions of microchannels are varied as described in the Results and Discussion section. For all cells, the nominal wall thickness λ^nom^ is set to 0.90 mm. All cells are supported with a base (height 3.1 mm). All microchannels are covered with a bridge (height 1.5 mm) to prevent their mechanical deformation. To mount electrodes, cells contain a groove at each end. A gap of 0.2 mm is designed between the cell and electrodes for easy operation. Electrodes have a handle for making a contact with a metallic crocodile clip of the measurement circuit (scheme in the inset of Figure 1). The thickness of the electrode and the handle is constant (2.5 mm).

## Results and Discussion

### Measurements of the Electrolyte Conductivity

Determination of κ performed in this work is based on measuring free currents in samples subjected to the alternating electric field. The theoretical background is presented in the Supporting Information. The functionality of the platform is demonstrated by employing aqueous 0.1 mol kg^–1^ KCl solution selected as the model electrolyte. KCl is available in high purity as a solid substance, which allows well-defined solutions with an exact concentration to be prepared. Furthermore, aqueous 0.1 mol kg^–1^ KCl has a well-documented temperature dependence of κ.^50^ The determination of κ was performed utilizing reference cells (Figure 1) with varied lengths (see the Experimental Section). Data obtained in reference cells were further employed to determine the resistance of sensing electrodes.

The impedance response was first measured using a commercial potentiostat (see the Supporting Information for details) to provide control data for subsequent inspections performed on the electronic platform used further (Figure 1 inset). Measurements using the potentiostat were carried out at the temperature of 26.5° C. Obtained impedance spectra are shown as Bode plots in Figure S1A. In the low-frequency range, |*Z*| values decrease with increasing frequency, reaching constant values at the high end. Such a profile is characteristic for a Maxwell–Wagner relaxation of the electrode/electrolyte system. At low frequency values, the response is dominated by capacitive reactance originating from charging of electric double layers at the two electrode/electrolyte interfaces (each of them having the capacitance denoted as *C*_dl_, see the equivalent circuit in Figure S1B). At higher frequency, both double layers are short-circuited, allowing properties of the medium introduced to the cell (represented by parallel *R*_cell_ and *C*_cell_ elements) to be sensed. Constant |*Z*| values obtained at high frequencies imply that the *C*_cell_ contribution is negligible for the 0.1 mol kg^–1^ KCl electrolyte. This allows values of *R*_cell_ to be extracted from the high frequency limit of |*Z*|, which equals *R* = *R*_cell_ + 2*R*_el_, where 2*R*_el_ is the resistance of electrodes. The *C*_dl_ values were inferred from the Maxwell–Wagner relaxation frequency (denoted as *f*_tr_ in Figure S1A) as *C*_dl_ = 1/π*f*_tr_*R* and fall within the range between 0.5 and 1 μF. As expected, high frequency limits of |*Z*| increase with the cell length. In the absence of capacitive contributions, the slope ∂|*Z*|/∂*l*_R_ equals 1/*A*_R_κ (see eq S9 in the Supporting Information), where *A*_R_ is the cross-sectional area of the cell (4 × 10^–4^ m^2^). The obtained value (1.86 kΩ/m) is identical for 5, 10, and 100 kHz (Figure S1B), further corroborating that the *C*_cell_ contribution is insignificant in the probed system. The value of the slope translates to the κ value of 1.34 S m^–1^, which is in a perfect agreement with the value of κ of 1.32 S m^–1^ obtained for aqueous 0.1 mol kg^–1^ KCl at 26.5 °C from the NIST database.^50^ The intercept in the |*Z*| versus *l*_R_ dependence at the highest probed frequency (100 kHz) estimates the 2*R*_el_ value and amounts to 70 Ω (Figure S1B).

**Figure 2:**
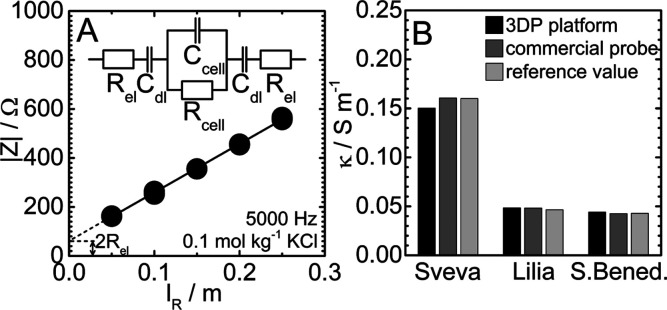
(A) |*Z*| values obtained at 5 kHz in reference cells of varied length *l*_R_ filled with the aqueous 0.1 mol kg^–1^ KCl electrolyte at 23.5 °C. (B) Conductivity values of bottled water obtained in this work using the 3DP platform (black) and commercial probe (dark gray) and values declared by the producer (light gray).

In all subsequent work, the potentiostat was replaced by the measurement platform composed of the function generator, the ammeter, and the voltmeter (inset of Figure S1, Supporting Information for details). The setup functionality was first verified by inspecting the impedance response of resistors with nominal *R*_nom_ values ranging between 10^1^ and 10^6^ Ω (covering resistance values expected further in this work) and a perturbation frequency of either 5 or 10 kHz. The output amplitude of the function generator was adjusted so that the resulting voltmeter reading (*U*_rms_) was in the range between 280 and 285 mV (corresponding to the *U*_0_ value of 200 mV). The measurable range of |*Z*| values is terminated at 1.5 × 10^5^ Ω (see Figure S2A), where current values (*I*_rms_) reach the lower detection limit of the ammeter (≈2 μA). Importantly, the electronic platform has a linear |*Z*| versus *R*_nom_ response spanning 4 orders of magnitude. The measurement accuracy (expressed as |*Z*|/*R*_nom_, inset of Figure S2A) is close to unity up to 1 × 10^5^ Ω. Deviations observed at higher *R*_nom_ values are ascribed to the parasitic capacitive reactance of cables (not considered in the equivalent circuit). Due to superior accuracy, the 5 kHz frequency was selected further in the conductometric analysis of electrolytes. Figure 2A shows |*Z*| values of reference cells filled with aqueous 0.1 mol kg^–1^ KCl at 23.5 °C. The found |*Z*| on *l*_R_ dependence corresponds well to that obtained using the commercial potentiostat (Figure S1B). The resulting ∂|*Z*|/∂*l*_R_ slope translates itself to the κ value of 1.26 S m^–1^, which is in a perfect agreement with the value obtained from the NIST database (1.25 S m^–1^ at 23.5 °C).^50^ The value of 2*R*_el_ amounts to 62 Ω, which is very close to that obtained using the potentiostat (70 Ω, Figure S1B).

The developed 3DP platform was further employed to determine κ values of bottled water samples (brands Sveva, Lilia, and San Benedetto) utilizing the approach presented for the KCl electrolyte. Results are plotted in Figure 2B together with values obtained using a commercial conductometric probe (see the Supporting Information for details) and reference values declared by water producers. In all cases, results obtained in the 3DP platform show a relative deviation of less than 6% compared to the commercial probe and declared values, demonstrating a high measurement accuracy.

It is worth noting that the transition frequency depends on the cell geometry and conductivity of inspected media. In the current cell design, the frequency of 5000 Hz is applicable for sensing all media with a conductivity comparable to and lower than that of aqueous 0.1 mol kg^–1^ KCl. For very concentrated electrolytes, the working frequency would have to be increased to obtain accurate results. In new cell designs, the frequency scan must always be performed, with the working frequency being then set sufficiently above the Maxwell–Wagner relaxation frequency.

### Measurements of the Dielectric Permittivity

Determination of ϵ_r_ performed in this work is based on measuring displacement currents in samples subjected to the alternating electric field. The theoretical background is presented in the Supporting Information. The functionality of the platform is demonstrated employing water, ethanol, and their mixtures with systematically varied molar ratios selected as model dielectrics. To maximize the current signal, the reference cell with *l*_R_ reduced to 0.01 m (see Figure 1) was employed in all ϵ_r_ measurements. The setup functionality was first verified by inspecting the impedance of capacitors with nominal *C*_nom_ values of 5.1, 12, 33, and 56 pF, corresponding to the expected response of common polar dielectric liquids introduced to the cell.

Impedance spectroscopy measurements were carried out at a *U*_rms_ of 283 mV (*U*_0_ value of 200 mV) and a frequency ranging from 5 to 100 kHz (increments of 5 kHz). To quantify the parallel parasitic capacitance of cables (*C*_p_, see the equivalent circuit in Figure S2B), impedance measurements were first performed without capacitors. The observed parasitic currents (denoted as *I*_p,rms_) were in the microampere range (data not shown) and, as expected, scaled with frequency. *C*_p_ values were obtained as *I*_p,rms_/*U*_rms_ω and average to 57 ± 4 pA. Subsequently, measurements were repeated with capacitors connected to the circuit, obtaining the response *I*_rms_. The impedance of capacitors |*Z*_c_| was determined as *U*_rms_/(*I*_rms_ – *I*_p,rms_). For *f* < 20 kHz, the obtained values of *I*_p,rms_ were below the lower detection limit of the ammeter (≈2 μA), hampering further analysis. For 20 < *f* < 70 kHz, the obtained impedance plotted as 1/|*Z*_c_| scales linearly with ω (Figure S2B), and the experimental capacitance *C*_c_ was determined as the slope of the best linear fit ∂(1/|*Z*_c_|)/∂ω. For *f* > 70 kHz, deviations from linearity were noticed (data not shown and considered further), presumably due to the reduced accuracy of the current and/or voltage sensing. For all capacitors, the found *C*_c_ values are very close to their respective *C*_nom_ values (deviations less than 5%), confirming that the developed platform senses displacement currents with very high accuracy.

**Figure 3:**
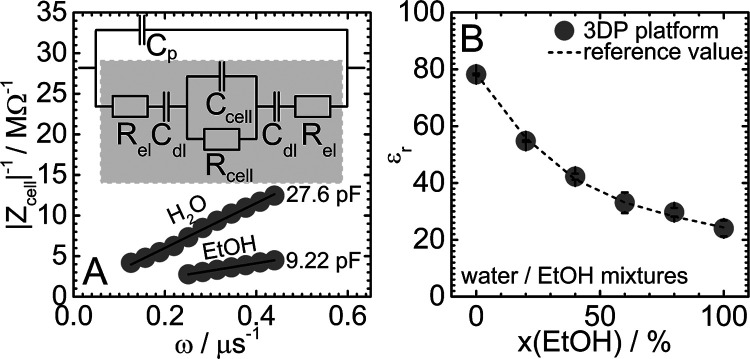
(A) Values of 1/|*Z*_cell_| as a function of angular frequency ω obtained for the reference cell (*l*_R_ = 0.01 m) filled with water and ethanol at 25 °C. (B) Values of ϵ_r_ of water/ethanol mixtures as a function of the molar fraction of ethanol x(EtOH).

Measurements of ϵ_r_ of water, ethanol, and their mixtures followed the approach presented for capacitors. Parasitic currents were recorded in the empty cell connected to the measurement circuit, with the frequency ranging from 20 to 70 kHz. The scan was subsequently repeated for the cell filled with the respective liquid (all measurements were performed at 25 °C). The correction for the parasitic capacitance *C*_p_ was performed, obtaining |*Z*_cell_| values on the order of 10^5^ Ω. This implies that impedance contributions of serial *R*_el_ and *C*_dl_ element pairs (both below 100 Ω as demonstrated above) may be ignored in the selected frequency range. The *C*_dl_ elements would cause noticeable contributions to the impedance magnitude only in the frequency range much lower than that utilized in ϵ_r_ measurements (see Figure S1A). Figure 3A shows values of 1/|*Z*_cell_| as a function of ω obtained for water and ethanol together with the proposed equivalent circuit (*Z*_cell_ is denoted by the gray area). The best linear fits to the 1/|*Z*_cell_| versus ω dependence show close-to-zero intercepts (*R*_cell_ → ∞), as expected for dielectrics. Values of *C*_cell_ were obtained as slopes ∂(1/|*Z*_cell_|)/∂ω and converted to ϵ_r_ values based on cell dimensions (see the Experimental Section). Results are plotted as a function of the molar fraction of ethanol, x(EtOH), as full circles in Figure 3B together with ϵ_r_ values reported by Moriyoshi et al. (dashed curve).^51^ For all mixtures, the deviation between our and reported data is less than 5%.

It is important to mention that PLA selected in this work as the material to manufacture cells and electrodes has limited resilience to organic solvents.^52^ The presented measurement approaches may be extended to the analysis of these liquids only when more inert materials are utilized. While our recent contribution^53^ has clearly demonstrated that cells printed from polyamide are resistant to 1,2-dichloroethane (ϵ_r_ = 10.4), electrically conductive composites based on polyamide are, to the best of our knowledge, not yet commercially available.

### Structural Investigation of 3D Printed Microchannels by Impedance Measurements

**Figure 4:**
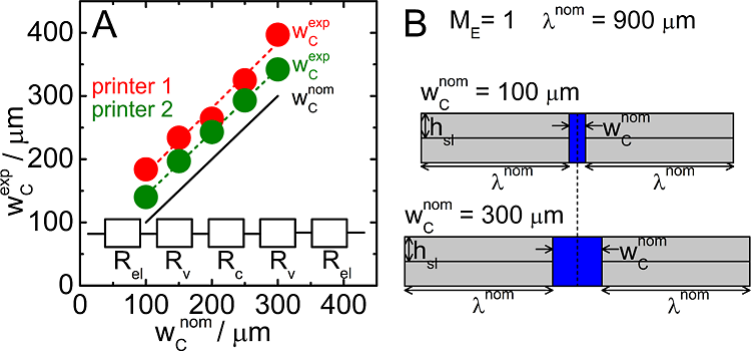
(A) Values of the experimental microchannel width (*w*_c_^exp^) obtained from impedance measurements performed at 5 kHz in cells filled with the aqueous 0.1 mol kg^–1^ KCl electrolyte plotted as a function of the nominal width (*w*_c_^nom^). Cells were manufactured employing printers 1 (red) and 2 (green) with an extrusion multiplier *M*_E_ of 1.00, where the microchannel length (*l*_c_) is 0.05 m. (B) Schematic cross-sectional depiction of microchannels with varied *w*_c_^nom^ (λ^nom^ = 900 μm and *h*_sl_ = 150 μm denote the nominal wall thickness and single layer height, respectively).

The results presented above demonstrate that the developed 3DP platform composed of the cell and electrodes with known dimensions allows for accurate and precise impedimetric sensing of liquid samples. Conversely, one can utilize impedance measurements employing liquids with known properties to sense dimensions of unknown structures. We apply this concept to investigate the width of 3DP microchannels terminated by two vessels (see Figure 1) serving for the introduction of the sensing electrolyte (aqueous 0.1 mol kg^–1^ KCl solution). Cells were manufactured employing two independent 3D printers (denoted as 1 and 2, see the Supporting Information). The minimum achievable microchannel width and the deviation between the real width and the desired (nominal) width are further considered as measures of the resolution and accuracy of employed printing protocols. All impedance measurements were performed at the perturbation frequency of 5 kHz with the *U*_rms_ value ranging between 280 and 285 mV (*U*_0_ value of 200 mV). Due to the designed geometry, the *R*_cell_ term may be split into the *R*_c_ term and the 2*R*_v_ term, accounting for the electrolyte resistance in the microchannel and vessels. The equivalent circuit (see Figure 4A) further involves resistive contributions of electrodes (2*R*_el_). As demonstrated above, impedance terms due to double layer charging and dielectric polarization of water molecules (present in the sensing electrolyte) are insignificant at the selected frequency, and the respective elements may be thus omitted. Taking this model, the microchannel width *w*_c_^exp^ obtained based on impedance magnitude measurements is
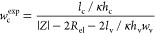
1where κ is the conductivity of the sensing electrolyte. For all microchannels manufactured in this work, the two surrounding vessels have constant dimensions (*l*_v_ = 0.05 m and *h*_v_ = *w*_v_ = 0.02 m). The height of microchannels *h*_c_ is constant (0.02 m), while their length *l*_c_ varies from 0.03 to 0.07 m (see below for details). Measurements using the Vernier caliper revealed that real and nominal values of *l*_v_, *h*_v_, *w*_v_, *h*_c_, and *l*_c_ differ by at most 1%, which allows respective nominal values to be introduced to eq 1.

In the first measurement campaign, microchannels with a constant *l*_c_ value of 0.05 m and a nominal width *w*_c_^nom^ varied from 100 to 300 μm (Figure 4B) were inspected. Structures with a *w*_c_^nom^ less than 100 μm could not be manufactured due to limitations of the employed slicing software.

**Figure 5:**
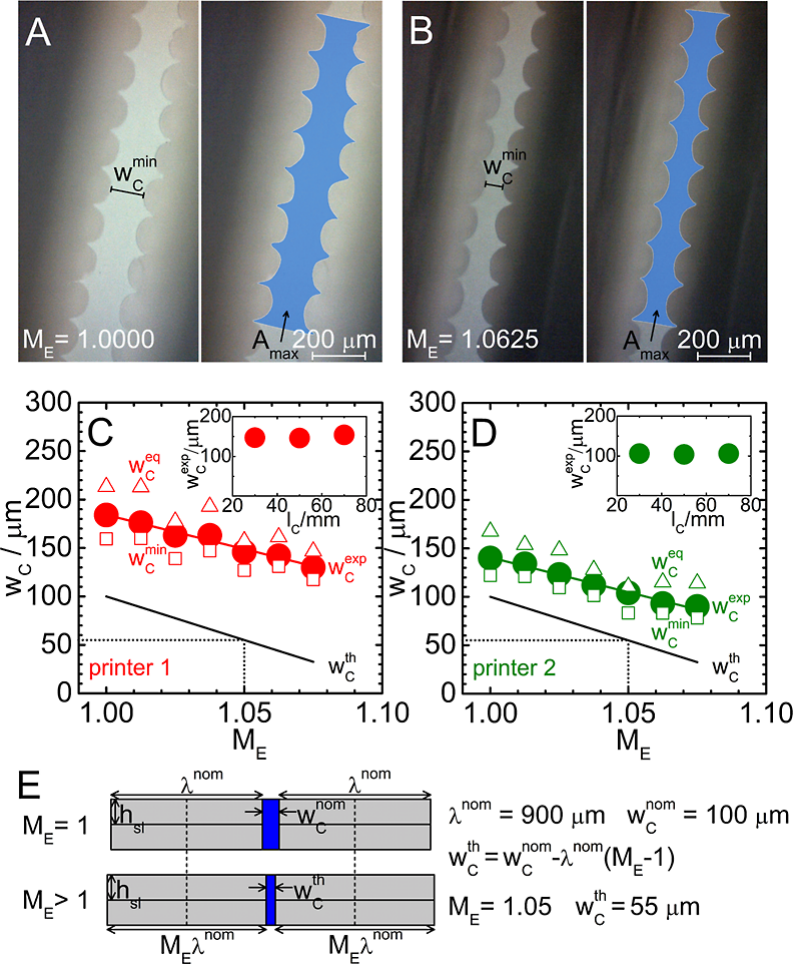
(A,B) Representative cross-sectional optical microscopic images of silicon imprints originating from microchannels with *w*_c_^nom^ = 100 μm and *l*_c_ = 0.05 m manufactured using printer 2 with the *M*_E_ of 1.0000 (A) and 1.0625 (B). (C,D) Averaged values of *w*_c_^exp^ (circles) obtained from impedimetric measurements and *w*_c_^min^ (squares) and *w*_c_^eq^ (triangles) extracted from optical microscopic imaging as a function of *M*_E_ for microchannels manufactured using printers 1 and 2. Black lines represent the theoretical microchannel width *w*_c_^th^ as a function of *M*_E_ calculated based on the model presented in (E). Insets in (C,D) show values of *w*_c_^exp^ obtained for microchannels with *w*_c_^nom^ = 100 μm and varied *l*_c_ printed with *M*_E_ = 1.05.

Figure 4A shows the *w*_c_^exp^ values determined from impedance measurements as a function of *w*_c_^nom^. For both employed printers, *w*_c_^exp^ values are systematically higher than *w*_c_^nom^ values, with the best linear fits lying almost parallel (∂*w*_c_^exp^/∂*w*_c_^nom^ slopes of 1.03 and 1.01) to the theoretical prediction (black line). Intercepts found by extrapolating fits to *w*_c_^nom^ = 0 reflect the manufacturing accuracy, δ*w*_c_ = *w*_c_^exp^ – *w*_c_^nom^. The obtained values amount to 74 and 43 μm. Literature studies comparing experimentally measured and nominal widths of microchannels manufactured by FDM-3DP all agree on close-to-unity ∂*w*_c_^exp^/∂*w*_c_^nom^ values but substantially differ in reported δ*w*_c_ values (−114 to 250 μm).^46,54−58^ The high scattering of δ*w*_c_ values suggests that dimensions of microchannels are sensitive to printing conditions. While their optimization was not the goal of our work, special attention was paid to avoiding printing artifacts (elephant feet, warping, under-extrusion, and over-extrusion). For a list and management of printing artifacts, we refer the interested reader to the PrintaGuide web page.^59^ The second measurement campaign was aimed at reducing and fine-tuning the microchannel width, focusing on those with a *w*_c_^nom^ value of 100 μm. Cells were printed with a systematically varied amount of the deposited material by tuning the extrusion multiplier (*M*_E_) value in the range between 1.000 and 1.075. Values greater than 1.075 led to over-extrusion and hence issues with filament loading and were thus avoided. Impedimetric characterization of microchannels was complemented by optical microscopic imaging of silicon imprints hardened in their interior (see the Supporting Information for details), which provided independent information about their width. Figure 5A,B shows representative crosssectional optical microscopic images of silicon imprints originating from microchannels printed with *M*_E_ values of 1.0000 (A) and 1.0625 (B). As expected, the increase of the *M*_E_ value leads to the narrowing of microchannels. Periodic patterns in the surface texture are due to the layer-by-layer nature of the 3DP. In each segment of the imprint, the minimum distance between wall surfaces, *w*_c_^min^, was evaluated. Additionally, the total (maximal) area *A*_max_ of the cross-section was obtained for a segment of imaged layers (denoted by blue area) by means of numerical integration. This obtained *A*_max_ value was divided by the single layer height (150 μm) and by the number of layers involved in the segment, yielding the equivalent width of the cross-section (*w*_c_^eq^).

Figure 5C,D shows values of *w*_c_^min^ (squares) and *w*_c_^eq^ (triangles) obtained as the average of characteristics extracted from three separately imaged cross-sections of a given microchannel further averaged over two independent microchannels. Circles depict averaged *w*_c_^exp^ values resulting from impedimetric characterization of two independent microchannels. The higher scattering of *w*_c_^min^ and *w*_c_^eq^ data points compared to more uniform *w*_c_^exp^ trends is ascribed to the local character of the optical microscopic analysis. As expected, all three characteristics decrease with increasing *M*_E_ values. We further derive and present a theoretical relation between the microchannel width and *M*_E_ employing a simple geometrical model shown in Figure 5E. The value of *M*_E_ reflects the relative flow rate of the liquefied filament extruded via the nozzle, with *M*_E_ = 1 being a reference value denoting the flow rate that leads to 100% solidity walls with desired (nominal) dimensions. For all microchannels in this work, the nominal wall thickness (λ^nom^) was set uniformly to 900 μm. Assuming that solid and liquefied filaments are incompressible, an increased amount of the extruded material (for *M*_E_ > 1) translates itself to the increase of the wall thickness (from λ^nom^ to *M*_E_λ^nom^). The wall broadening is considered as symmetrical (see Figure 5E). Under these conditions, the theoretical microchannel width at given *M*_E_, *w*_c_^th^, must satisfy the relation *M*_E_λ^nom^ + *w*_c_^th^ = λ^nom^ + *w*_c_^nom^, implying that ∂*w*_c_^th^/∂*M*_E_ = −λ^nom^. For λ^nom^ = 900 μm, the increase of the *M*_E_ value by 1% reduces the *w*_c_^th^ value by 9 μm. Figure 5E depicts the final formula relating *M*_E_ and *w*_c_^th^ and shows an illustrative calculation performed for λ^nom^ = 900 μm, *w*_c_^nom^ = 100 μm, and *M*_E_ = 1.05. The result of this calculation (55 μm) is depicted by dotted black lines in Figure 5C,D, and the complete *w*_c_^th^ versus *M*_E_ dependence is shown as a solid black line. Slopes of the best linear fits to *w*_c_^exp^ versus *M*_E_ data sets resulting from impedimetric measurements (red and green lines) amount to −7.1 and −7.2 μm/% and are thus close to the theoretical value. Experimental and theoretical values of ∂*w*_c_/∂*M*_E_ obtained in this work are close to the value of −10 μm/% found experimentally in the work of Mehta et al.^49^

Noteworthily, the *w*_c_^exp^ versus *M*_E_ dependence (Figure 5C,D) shows similar features to the *w*_c_^exp^ versus *w*_c_^nom^ plot (Figure 4A), that is, slopes of experimentally observed trends consistent with theoretical predictions but positive absolute deviations. Interestingly, for both printers and the entire *M*_E_ range inspected, *w*_c_^exp^ values lie between the respective *w*_c_^min^ and *w*_c_^eq^ values. This suggests that the central regions of microchannels (corresponding to *w*_c_^min^ values, Figure 5A,B) are completely filled with the sensing electrolyte, while side regions (accounted for only in *A*_max_ values) are filled only partially. Importantly, all *w*_c_^min^ values are systematically higher than the corresponding *w*_c_^th^ values, indicating that absolute deviations observed between *w*_c_^exp^ and *w*_c_^th^ data sets cannot be explained solely by the texture of microchannel walls. Therefore, the explanation of observed deviations must consider other peculiarities of FDM-3DP. These involve cohesion between the extruded liquefied filament and the underlying already solidified layers and the adhesion of the liquefied filament to the printing nozzle surface. Unequal δ*w*_c_ values found for the two printers in this work (74 vs 43 μm) could be rationalized by the varied interplay of these two factors. Changes in the width and surface texture of microchannels manufactured by FDM-3DP may also originate from the varied distance and adhesion between the first layer and the printing pad.^46^ For the *M*_E_ value of 1.05, we have additionally printed and inspected microchannels with the *l*_c_ value being changed from 0.05 to 0.03 and 0.07 m. As expected, values of *w*_c_^exp^ were found to be independent of *l*_c_ (insets in Figure 5C,D). This observation corroborates the correctness of the theoretical model built to describe the electric response of cells with microchannels (eq 1 and the equivalent circuit in Figure 4A) and implies that findings obtained in this work may be generalized to microchannels of varied length.

Systematic trends found for microchannels in this work demonstrate that FDM-3DP enables the manufacture of microfluidic architectures with well-controlled dimensions. Absolute deviations between experimental and theoretical (nominal) widths may be minimized by altering the extrusion multiplier value. We successfully printed microchannels with the width down to 80 μm. This value represents a better manufacturing resolution than that achieved by FDM-3DP in other reported studies,^37,45,47,49,54−58^ with the only exception being that of Nelson et al.^46^ (width of 40 μm). Our work introduces a non-destructive impedimetric approach for sensing microfluidic architectures. To get deeper insights into electric sensing in 3DP microfluidic channels, we envisage to implement computational modeling of mass and charge transport phenomena.^60^ Approaches developed in this work will be utilized in the manufacture of 3DP lab-on-a-chip platforms for sensing viruses^61,62^ and cells.^63^

## Conclusions

The combination of CAD and bimaterial FDM-3DP was applied to devise and manufacture an integrated platform for measuring electric properties of liquids. The functionality of the platform was demonstrated by measuring conductivity of aqueous KCl solution and bottled water samples and permittivity of water, ethanol, and their mixtures. In all cases, the obtained results are in a perfect agreement with reference values.

Impedimetric analysis was further applied to investigate the width of 3D printed microchannels integrated to cells, employing aqueous KCl solution as the sensing electrolyte. The interior of microchannels was independently inspected by optical microscopy imaging of hardened silicone rubber imprints. Such a combined approach was employed to scrutinize microchannels printed with varied extrusion multiplier values. The developed printing protocol enabled the microchannel width of 80 μm to be reached, being one of the best achieved resolutions reported in the literature.

The presented impedimetric sensing of liquids and microfluidic architectures opens up new venues for micro-analytical methods. The analysis is non-destructive, requires no hazardous chemicals, and relies on basic physical principles that enable uncomplicated conversion of impedance data to the target material and structural characteristics. The employed manufacturing and sensing approaches are based on easy-to-operate and inexpensive equipment (the FDM 3D printer and basic electronic measurement setup). Importantly, the presented analysis may be extended to inspect the functionality of active microfluidic elements involving pumps, valves, or mixers. We envision that near-future advances in FDM-3DP will enable the manufacture of functional microfluidic elements and integrated devices.

## Supporting Information Available

Theoretical background of the work, procedures used for the printing of cells and electrodes, the preparation of liquid samples, impedance sensing of liquids and microchannels, and microscopic characterization of microchannels (PDF)
Assembly of the platform (MP4)
Steps performed for the assembly of the platform (PDF)
