# Supplementary material for: 3D Printed Platform for Impedimetric Sensing of Liquids and Microfluidic Channels
Source: Anal Chem. 2022 Oct 6;94(41):14426–33. doi: 10.1021/acs.analchem.2c03191 (PMC9951178; doi:10.1021/acs.analchem.2c03191)
Supplement: Supplementary file 1 — ac2c03191_si_001.pdf [file ac2c03191_si_001.pdf]

Supporting Information
3D printed platform for impedimetric sensing of liquids and microfluidic channels

Táňa Sebechlebská,† Eva Vaněčková,‡ Marta Katarzyna Choińska-Młynarczyk,‡ Tomáš Navrátil,‡ Lukasz Poltorak,¶ Andrea Bonini,§ Federico Vivaldi,*,§ and Viliam Kolivoška*,‡

†*Department of Physical and Theoretical Chemistry, Faculty of Natural Sciences, Comenius University in Bratislava, Mlynska Dolina, Ilkovicova 6, 84215 Bratislava 4, Slovakia*

‡*J. Heyrovsky Institute of Physical Chemistry of the Czech Academy of Sciences, Dolejskova 3, 18223 Prague, Czech Republic*

¶*Department of Inorganic and Analytical Chemistry, Faculty of Chemistry, University of Lodz, Tamka 12, 91-403 Lodz, Poland*

§*Department of Chemistry and Industrial Chemistry, University of Pisa, via Giuseppe Moruzzi 13, 56124 Pisa, Italy*

E-mail: federicomaria.vivaldi@phd.unipi.it; viliam.kolivoska@jh-inst.cas.cz

Table of Contents

Theoretical background of the work **S2**

Printing of cells and electrodes, assembly of the measurement setup **S5**

Sample preparation **S7**

Impedance spectroscopy measurements S8

Microscopic inspection of micro-channels S8

Impedance measurements by commercial potentiostat S9

Verification of the electronic sensing platform functionality by resistors and capacitors S10

References S11

Theoretical background of the work

The determination of electric conductivity κ and dielectric permittivity ϵ_r of materials is based on measuring electric response of their samples with known dimensions subjected to the external electric field $\mathbf{E}$. The conversion of measured current signal to κ and ϵ_r values requires a description of fundamental charge transport mechanisms in materials and in a vacuum as their underlying medium. A free current I_f refers to a flow of charged particles. In this work, free currents resulting from electric field $\mathbf{E}$ are considered only. When vacuum or material samples are exposed to time-dependent electric fields, currents also appear in relation to the charge separation. In materials, electric and magnetic dipoles exist due to the microscopic movement of electric charges. All these contributions sum up to the total current I_T, which, when expressed as the density $\mathbf{j}_T$, represents an observable linked to the generation of a magnetic field $\mathbf{B}$ as described by the Ampere-Maxwell law

$$\frac{1}{\mu_0}(\nabla \times \mathbf{B}) = \mathbf{j}_T = \mathbf{j}_f + \epsilon_0 \frac{\partial \mathbf{E}}{\partial t} + \frac{\partial \mathbf{P}}{\partial t} + \nabla \times \mathbf{M} \qquad (1)$$

Here, ϵ_0 and μ_0 is permittivity and permeability of the vacuum and $\mathbf{P}$ and $\mathbf{M}$ is volumetric density of electric and magnetic dipoles in the material. For isotropic electrically conductive materials, a constitutive relationship (Ohm‘s law) states that the free current density $\mathbf{j}_f$ is

proportional to and aligned with the applied electric field

$$\mathbf{j_f} = \kappa \mathbf{E} \tag{2}$$

with κ being electric conductivity of the material. In dielectrics subjected to the electric field, permanent electric dipoles within their particles (if present) are aligned towards the direction of the field and induced electric dipoles are formed by microscopic separation of charges (atomic nuclei and electron shells). For homogeneous isotropic dielectric materials with no memory effects a constitutive relationship holds between $\mathbf{P}$ and $\mathbf{E}$

$$\mathbf{P} = \varepsilon_0 \chi \mathbf{E} \tag{3}$$

where χ is the electric susceptibility of the material. Contributions to $\mathbf{j}_T$ due to the charge separation and dielectric polarization in the material are treated collectively as the displacement current density $\mathbf{j}_D = \partial \mathbf{D}/\partial t$, where $\mathbf{D}$ is the displacement field

$$\mathbf{D} = \varepsilon_0 \mathbf{E} + \mathbf{P} = \varepsilon_0 \mathbf{E} + \varepsilon_0 \chi \mathbf{E} = \varepsilon_0 (1 + \chi) \mathbf{E} = \varepsilon_0 \varepsilon_r \mathbf{E} \tag{4}$$

and $\epsilon_r = 1 + \chi$ is the relative permittivity of the material. In general, susceptibility and permittivity are mathematically complex quantities with the imaginary part reflecting energy losses due to rotation of electric dipoles. However, in liquids investigated in this work, the only dipoles are that of water and ethanol molecules which have their rotational diffusion time scales in GHz range. In the range of kHz explored in this work, the imaginary part of permittivity due to this effect is negligible. Investigated liquids are also intrinsically non-magnetic and no external magnetic field is applied in performed experiments implying that no permanent and induced magnetic dipoles are present in investigated systems ($\mathbf{M} = \mathbf{0}$).

Under these conditions, the total current density is

$$\mathbf{j_T} = \kappa \mathbf{E} + \varepsilon_0 \varepsilon_r \frac{\partial \mathbf{E}}{\partial t} \tag{5}$$

In this work, harmonic voltage $U = U_0 sin(\omega t)$ is applied between parallel-plate electrodes in rectangular reference cells (their length and cross-sectional area are denoted as l_R and A_R, see Fig. 1) completely filled with homogeneous media. The resulting electric field in the cell is harmonic $\mathbf{E} = \mathbf{E_0} sin(\omega t)$, homogeneous and its magnitude $|\mathbf{E}|$ may be expressed as U/l_R. Its rate of change is

$$\frac{\partial \mathbf{E}}{\partial t} = \omega \mathbf{E_0} cos(\omega t) = \omega \mathbf{E_0} sin\left(\omega t + \frac{\pi}{2}\right) = j\omega \mathbf{E_0} sin(\omega t) = j\omega \mathbf{E} \tag{6}$$

where $j^2 = -1$. The total current density is

$$\mathbf{j_T} = \kappa \mathbf{E} + j\omega \varepsilon_0 \varepsilon_r \mathbf{E} = (\kappa + j\omega \varepsilon_0 \varepsilon_r) \mathbf{E} \tag{7}$$

Its magnitude is

$$|\mathbf{j_T}|^2 = \mathbf{j_T}\widetilde{\mathbf{j_T}} = (\kappa + j\omega \varepsilon_0 \varepsilon_r) \mathbf{E} (\kappa - j\omega \varepsilon_0 \varepsilon_r) \widetilde{\mathbf{E}} = (\kappa^2 + \omega^2 \varepsilon_0^2 \varepsilon_r^2)|\mathbf{E}|^2 \tag{8}$$

In the rectangular geometry filled with an isotropic medium, $\mathbf{j}_T$ is homogeneous and $|\mathbf{j}_T|$ may be expressed as I_T/A_R. The total current is

$$I_T^2 = I_f^2 + I_D^2 = \left(\frac{UA_R}{l_R}\right)^2 (\kappa^2 + \epsilon_0^2 \epsilon_r^2 \omega^2) \tag{9}$$

In this work, root-mean square values of I_T and U (denoted as I_{rms} and U_{rms}) are sensed by dedicated electronics and converted to impedance magnitude $|Z| = U_{rms}/I_{rms}$. Contributions of free and displacement currents are separated by performing measurements at systematically varied values of ω (impedance spectroscopy). In the data analysis, we uti-

lize the concept of equivalent circuits with resistor and capacitor reflecting κ and ϵ_r as $R = l_R/\kappa A_R$ and $C = \epsilon_0 \epsilon_r A_R/l_R$.

Printing of cells and electrodes, assembly of the measurement setup

Altogether three FDM 3D printers (Prusa I3 MK3) were employed in this work. Cells were manufactured by two printers, both equipped with a brass extrusion nozzle with the inner diameter of 0.25 mm. The third printer with a brass extrusion nozzle with the inner diameter of 0.40 mm was used to make electrodes. All objects were printed on smooth polyetherimide-coated steel spring sheets (further referred to as printing pads). All 3D printers, extrusion nozzles and printing pads were purchased from Prusa Research, Czech Republic. Cells were printed from electrically insulating transparent polylactic acid (PLA) filament (1.75 mm diameter, Gembird, The Netherlands), while electrodes were printed from electrically conductive composite filament based on PLA and carbon nanotubes (PLA-CNT, 1.75 mm diameter, brand name FiloAlfa, AlfaOhm, Italy). Both kinds of filaments were stored in desiccators when not in use. Just before printing, the extruder of the printer was copiously purged with the respective filament at 250 °C to remove residues of previously utilized materials. The printing pad was repeatedly wiped with acetone (Penta, Czech Republic) at room temperature to remove ambient contamination. No chemical agents or tapes were used to modify the adhesion between printed objects and printing pads.

Geometries devised by CAD as described in the "Design of cells and electrodes" section were exported as high resolution stl files and processed in PrusaSlicer (version 2.0.0) software to generate gcode files containing all instructions for the printer. The slicing parameters were set as follows. For cells, the single layer height h_{sl} was set to 0.15 mm. For this value, the wall thickness of 0.90 mm and the nozzle diameter of 0.25 mm corresponds to four printed perimeters. For electrodes, h_{sl} was set to 0.20 mm. For this value, the wall thickness of

2.50 mm and the nozzle diameter of 0.40 mm corresponds to six printed perimeters. For electrodes and reference cells (for measuring conductivity and permittivity), the extrusion multiplier E_M was set to unity. For cells involving micro-channels, the E_M value was varied as described in the Results and Discussion section. For all printed objects, the temperature of the extruder and the printing pad was set to 225 and 60 °C, respectively. The printing speed value was set to 20 mm s^{-1} for the first layer, 25 mm s^{-1} for external perimeters, 45 mm s^{-1} for internal perimeters, 80 mm s^{-1} for the infill and 180 mm s^{-1} for non-printing moves. Retraction settings were set to default values for unmodified (insulating) PLA. All cells and electrodes were manufactured so that seams of individual layers were located beyond parts to be in contact with introduced liquid samples. Cell supports and top bridges of micro-channels were printed employing a rectilinear infill with 100 % solidity and angle of ±45°. Cells were printed in the bottom-up direction, while electrodes were printed in the top-down direction. For cells, an external brim with the width of 10 mm was added to the first printed layer (not shown in Fig. 1) to improve adhesion to the printing pad. No brim was used for electrodes. Upon manufacture, electrodes and cells were left to cool down to the room temperature and were gently separated from the printing pad by hand. Brims were removed by scissors. All printed objects were stored in closed glass containers when not in use to minimize ambient contamination.

Just before experiments, sides and the bottom surface of electrodes were covered with a single layer of 0.2 mm thick Teflon tape. Such masked electrodes were gently inserted to the groove at each side of the cell. Excess tape protruding to the cell interior was gently removed by a scalpel blade. This procedure was found to be sufficient to prevent leakage of liquids. No activation of electrodes was performed. A video depicting the assembly of the cell and its filling by the electrolyte (coloured by methylene blue for demonstration purpose) is available in a separate file.

Sample preparation

Deionized water (maximum total organic carbon of 3 ppb, minimum electric resistivity of 18.2 MΩ cm) was obtained by means of Millipore system, France. Absolute ethanol (99.8 %, p.a.) was obtained from Penta, Czech Republic. Solid KCl (puriss. p.a., 99.5 %) was obtained from Sigma Aldrich, Czech Republic. It was dried at 200 °C for 6 hours and stored in sealed glass bottles. Bottled water samples (brands Sveva, Lilia and San Benedetto) were purchased from the local store.

The applicability of the devised platform for sensing electric conductivity was inspected employing in-lab prepared aqueous 0.1 mol kg^{-1} (molality) KCl solution and reference cells with l_R ranging from 0.05 to 0.25 m. The platform was further utilized to measure the conductivity of bottled water samples. The electrolyte and water samples were introduced by a pipette to completely fill cells. Temperature resolved reference electric conductivity values of aqueous 0.1 mol kg^{-1} KCl were downloaded from the NIST database[1]. The conductivity of bottled water samples was independently inspected by a commercial probe (Delta Acque D202, Italy) in a glass beaker.

The applicability of the devised platform for sensing dielectric permittivity was demonstrated employing in-lab prepared mixtures of deionized water and absolute ethanol with molar fraction of ethanol being varied from 0 to 1 in increments of 0.2. Respective mixtures were prepared by weighing liquids. A reference cells with l_R of 0.01 m was employed in measurements. Mixtures were introduced by a pipette to completely fill the cell. Permittivity values obtained by the devised platform were compared to those reported for water/ethanol mixtures in the literature[2].

For measurements of micro-channel width, aqueous 0.1 mol kg^{-1} KCl solution was used as the sensing electrolyte. It was continuously introduced to one vessel of the cell and let to flow through the micro-channel to the other vessel until all three compartments were filled completely. A video depicting the assembly of the cell and its filling by the electrolyte (coloured by methylene blue for demonstration purpose) is available in a separate file.

Impedance spectroscopy measurements

Electrodes of the cell assembled and filled as described above were connected to the measurement circuit (inset of Fig. 1) by alligator clips. The measurement circuit consists of a commercial function generator (UTG1010A, purchased from UNI-T) serving as a source of harmonic (sine-wave) voltage and two commercial multimeters (UT71B, purchased from UNI-T) operating as an ammeter and a voltmeter. The impedance magnitude $|Z|$ was obtained as the ratio of root-mean square readings of the voltmeter U_{rms} and the ammeter I_{rms} and was evaluated as a function of frequency of applied voltage (impedance spectroscopy). The amplitude and frequency of applied voltage are specified in Results and Discussion section.

For aqueous 0.1 mol kg^{-1} KCl electrolyte, conductivity measurements were additionally carried out employing integrated electrochemical impedance spectroscopy module within the commercial potentiostat (Autolab, Metrohm, Czech Republic) at zero DC voltage, perturbation root-mean square amplitude of 10 mV and frequency ranging from 10^0 to 10^5 Hz. In these measurements, one electrode of the setup was connected to the potentiostat as the working electrode while the other one jointly as the reference and the counter electrode.

All experiments in this work were performed at ambient pressure and temperature ranging from 20 to 30 °C. The temperature of liquids was in particular experiments monitored with the precision of ±0.5 °C employing a conventional lab thermometer and is specified in Results and Discussion section. After measurements, cells and electrodes were copiously rinsed by deionized water and were left to dry in glass containers.

Microscopic inspection of micro-channels

The interior of micro-channels was inspected by optical microscopy imaging as follows. Freshly mixed liquid silicone rubber (Mold Star 15 SLOW, Smooth-On, USA) was introduced to one vessel of the cell. The cell was tilted by 45°, which allowed the micro-channel

to be filled by gravity. Upon hardening of silicone rubber, cells were gently disintegrated and imprints formed in micro-channels were separated from walls by tweezers. Imprints were cut perpendicularly by a scalpel blade to reveal their cross-sectional profiles. Such obtained profiles were inspected by an optical microscope (NMM800TR, Microteb) equipped with a camera (Dino-EYE AM4023CT). Dimensions and area of cross-sectional profiles were evaluated in the environment of Fusion 360.

Impedance measurements by commercial potentiostat

Fig. S1A shows impedance magnitude as a function of applied frequency measured by a commercial potentiostat in reference cells of varied length filled with aqueous 0.1 mol kg^{-1} KCl. Fig. S2B shows impedance magnitude evaluated at 5, 10 and 100 kHz vs. cell length dependence.

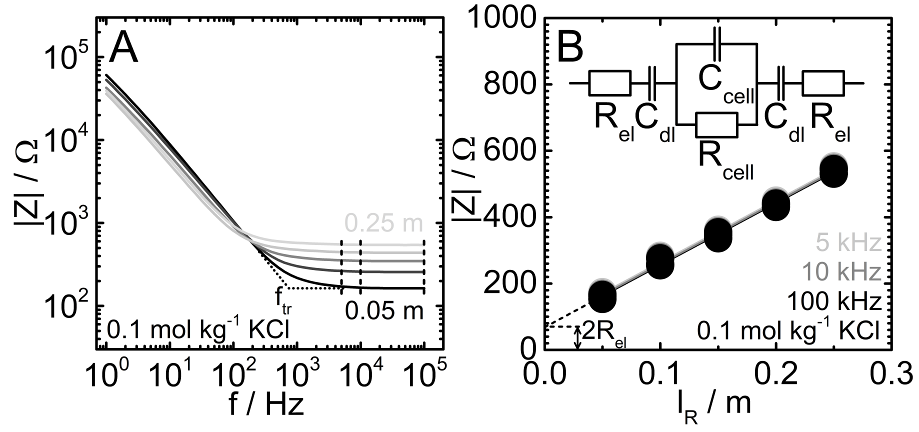

Figure S1: (A) Bode plots constructed for impedance magnitude $|Z|$ measured by a commercial potentiostat in reference cells with the length l_R ranging from 0.05 to 0.25 m (shades of gray) filled with aqueous 0.1 mol kg^{-1} KCl at 26.5 °C. (B) Dependence of $|Z|$ on l_R, with $|Z|$ being extracted at 5, 10 and 100 kHz (dashed lines in (A)).

Verification of the electronic sensing platform functionality by resistors and capacitors

Fig. S2A shows impedance magnitude obtained for resistors at 5 and 10 kHz as a function of their nominal resistance. The inset shows the measurement accuracy. Fig. S2B shows inverse impedance magnitude of capacitors vs. applied angular frequency.

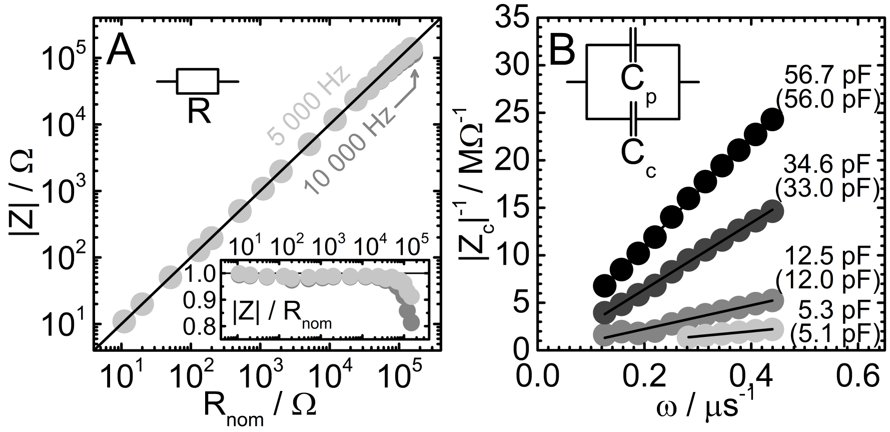

Figure S2: (A) Impedance magnitude $|Z|$ obtained for resistors at 5 kHz (light grey) and 10 kHz (dark grey) as a function of their nominal resistance value R_{nom}. The inset shows the measurement accuracy expressed as $|Z|/R_{nom}$. (B) Inverse impedance magnitude of capacitors $1/|Z_c|$ as a function of angular frequency ω. Experimental and nominal values of capacitance C_c are presented as numbers (the latter in brackets).

References

(1) R. Shreiner, K. Pratt, Standard reference materials: primary standards and standard reference materials for electrolytic conductivity, NIST Special Publication 260 (2004) 142.

(2) T. Moriyoshi, T. Ishii, Y. Tamai, M. Tado, Static dielectric constants of water + ethanol and water + 2-methyl-2-propanol mixtures from 0.1 to 300 MPa at 298.15 K, J. Chem. Eng. Data 35 (1990) 17-20. doi:https://doi.org/10.1021/je00059a005.
