# Supplementary material for: 3D Printed Platform for Impedimetric Sensing of Liquids and Microfluidic Channels
Source: Anal Chem. 2022 Oct 6;94(41):14426–33. doi: 10.1021/acs.analchem.2c03191 (PMC9951178; doi:10.1021/acs.analchem.2c03191)
Supplement: Supplementary file 3 — ac2c03191_si_003.pdf [file ac2c03191_si_003.pdf]

0 min 00 sec – 0 min 35 sec Masking one of electrodes with the teflon tape

0 min 35 sec – 0 min 45 sec Insering the electrode to the cell

0 min 45 sec – 1 min 45 sec Removing excess teflon tape

1 min 55 sec – 4 min 05 sec Introducing aqueous 0.1 mol kg-1 as the sensing electrolyte to the cell (the electrolyte is coloured by 1 mM methylene blue for better visualization in this video)

4 min 05 sec – 4 min 25 sec Connecting electrodes to the measurement circuit (function generator/microampermeter/millivoltmeter)
